# Transgene Optimization, Immunogenicity and *In Vitro* Efficacy of Viral Vectored Vaccines Expressing Two Alleles of Plasmodium falciparum AMA1

**DOI:** 10.1371/journal.pone.0020977

**Published:** 2011-06-16

**Authors:** Sumi Biswas, Matthew D. J. Dicks, Carole A. Long, Edmond J. Remarque, Loredana Siani, Stefano Colloca, Matthew G. Cottingham, Anthony A. Holder, Sarah C. Gilbert, Adrian V. S. Hill, Simon J. Draper

**Affiliations:** 1 The Jenner Institute, University of Oxford, Oxford, Oxfordshire, United Kingdom; 2 Laboratory of Malaria and Vector Research, National Institute of Allergy and Infectious Diseases/National Institutes of Health, Rockville, Maryland, United States of America; 3 Department of Parasitology, Biomedical Primate Research Center, Rijswijk, The Netherlands; 4 Okairòs AG, Rome, Italy; 5 Divison of Parasitology, National Institute for Medical Research, London, United Kingdom; World Health Organization, Switzerland

## Abstract

**Background:**

Apical membrane antigen 1 (AMA1) is a leading candidate vaccine antigen against blood-stage malaria, although to date numerous clinical trials using mainly protein-in-adjuvant vaccines have shown limited success. Here we describe the pre-clinical development and optimization of recombinant human and simian adenoviral (AdHu5 and ChAd63) and orthopoxviral (MVA) vectors encoding transgene inserts for *Plasmodium falciparum* AMA1 (PfAMA1).

**Methodology/Principal Findings:**

AdHu5-MVA prime-boost vaccination in mice and rabbits using these vectors encoding the 3D7 allele of PfAMA1 induced cellular immune responses as well as high-titer antibodies that showed growth inhibitory activity (GIA) against the homologous but not heterologous parasite strains. In an effort to overcome the issues of PfAMA1 antigenic polymorphism and pre-existing immunity to AdHu5, a simian adenoviral (ChAd63) vector and MVA encoding two alleles of PfAMA1 were developed. This antigen, composed of the 3D7 and FVO alleles of PfAMA1 fused in tandem and with expression driven by a single promoter, was optimized for antigen secretion and transmembrane expression. These bi-allelic PfAMA1 vaccines, when administered to mice and rabbits, demonstrated comparable immunogenicity to the mono-allelic vaccines and purified serum IgG now showed GIA against the two divergent strains of *P. falciparum* encoded in the vaccine. CD8^+^ and CD4^+^ T cell responses against epitopes that were both common and unique to the two alleles of PfAMA1 were also measured in mice.

**Conclusions/Significance:**

Optimized transgene inserts encoding two divergent alleles of the same antigen can be successfully inserted into adeno- and pox-viral vaccine vectors. Adenovirus-MVA immunization leads to the induction of T cell responses common to both alleles, as well as functional antibody responses that are effective against both of the encoded strains of *P. falciparum in vitro*. These data support the further clinical development of these vaccine candidates in Phase I/IIa clinical trials.

## Introduction

It is estimated that in Africa alone there are 200 million cases and 0.8 million deaths due to malaria every year [1]. Vaccines remain one of the most effective public health interventions to reduce morbidity and mortality. The worldwide eradication of smallpox was due to vaccination and other infectious diseases (such as poliomyelitis and rinderpest of cattle) are similarly on the brink of eradication. In the case of malaria the development of a highly effective vaccine remains a high priority. Vaccine-induced immunity in an endemic area could help to significantly reduce overall transmission by inducing herd immunity when combined with pre-existing partially effective control interventions including insecticide-treated bednets, artemisinin-based combination therapies and indoor residual spraying [2]. Blood-stage malaria vaccines aim to mimic immunity that is naturally acquired in malaria endemic areas [3]. Individuals living in malaria endemic countries develop immunity by repeated exposure to the parasite and a significant proportion of this immunity is directed to antigens expressed on the blood-stage parasites or infected erythrocytes. This immunity is mostly antibody mediated [4], although more recently the importance of cellular immunity has begun to be recognized in mice [5], [6] and humans [7], [8].

Blood-stage malaria subunit vaccine development has thus mainly focused on antibody-inducing protein-in-adjuvant formulations targeting well-studied merozoite antigens, including merozoite surface proteins (MSPs) and antigens secreted from apical organelles of the parasite during erythrocyte invasion, such as apical membrane antigen 1 (AMA1). To date there have been over 30 phase I/II clinical trials with these blood-stage antigens reported [3], but the limited success of such candidates, combined with reports of vaccine allele-specific efficacy in field trials [9] and in *in vitro* assays of purified IgG growth inhibitory activity (GIA) [10], [11], indicates that blood-stage vaccines may need to include multiple alleles of the same antigen to achieve significant efficacy against the many strains of *P. falciparum* in the field.

PfAMA1 has been one of the leading blood-stage malaria vaccine candidate antigens for a considerable time, and there have been numerous pre-clinical and clinical AMA1 vaccine studies (reviewed in Ref [12]). Field studies have primarily addressed the importance of antibodies to PfAMA1 to clinical immunity, showing that in naturally exposed individuals the prevalence of PfAMA1-specific IgG increases with age and that this is associated with reduced risk of clinical malaria [13], [14], [15]. However, the PfAMA1 antigen is polymorphic, probably as a result of immune selection operating on this important target of naturally occurring immunity, and antibodies raised against individual naturally-occurring alleles of this antigen inhibit growth of *P. falciparum* strains *in vitro* in a strain-specific manner. A clinical trial of an PfAMA1 3D7 allele protein vaccine (FMP2.1) showed that sera from vaccinees, although capable of inhibiting growth of 3D7 strain *P. falciparum* parasites *in vitro*, afforded no inhibition of the heterologous FVO strain [16].

Several different approaches have thus been developed to address the issue of polymorphism [3]. The AMA1-C1 recombinant protein vaccine in clinical development is composed of a mixture of the 3D7 and FVO alleles [17] – two of the most divergent in terms of polymorphism and which differ by 24 amino acids out of 622 amino acids. It has been shown that vaccination with multivalent or chimeric PfAMA1 vaccines increases the levels of antibodies to common allele epitopes and/or broadens inhibition of malaria strains [18], [19], [20]. Immunization with diversity-covering (DiCo) PfAMA1 sequences, that on average incorporate 97% of the amino acid variability observed in PfAMA1, has also been shown to induce broader functional immunity than immunization with mixtures of naturally-occurring alleles [21].

Conversely, studies of T cell-mediated immunity against PfAMA1 and/or blood-stage malaria infection in humans have been more limited. In one epidemiological study, seven T cell epitopes in PfAMA1 were reported, with some localized to conserved areas of PfAMA1 – an important observation in terms of vaccination strategy against this polymorphic antigen [22]. Individuals showing a proliferative response to one PfAMA1 peptide corresponding to aa 259–271 were found to be less likely to be parasitemic on follow up [23]. Another study has shown that repeated ultra-low dose exposure of malaria-naïve human volunteers to blood-stage parasites and drug cure can induce sterile protection against a blood-stage challenge. In this study there was no detectable antibody response against the parasite. A cell-mediated immune response was measured – mainly characterized by a proliferative CD4^+^ and CD8^+^ T cell response and Th1-type cytokine induction [7]. In another study, volunteers were protected against malaria challenge with infected mosquitoes after sporozoite inoculation and chloroquine drug cure, and parasite-specific pluripotent effector memory T cells before challenge were found to be associated with protection [8]. In rodent malaria models, there is strong evidence for the role of CD4^+^ T cells, interferon-gamma (IFN-γ) and nitric oxide contributing to the control of blood-stage parasitaemia [6], [24], including those cells against the AMA1 antigen [5]. Recently a study in the *P. chabaudi* AS model showed that the failure to maintain long-term protective responses was due to a gradual decline in the parasite-specific memory CD4^+^ T cell response, despite persistent B cell memory and circulating antibodies [25]. This study provides an important insight into T and B cell memory to malaria and encourages vaccination strategies that induce memory T cells to ensure long-term efficacy. With increasing evidence of the role for T cells, as well as antibodies, in blood-stage malaria immunity, vaccine development strategies should focus on vaccine platforms capable of generating both humoral and cellular immunity [26]. This strategy could induce a broader repertoire of immune responses to target such polymorphic malarial proteins.

Recently, replication-deficient recombinant viral vectored vaccination regimens have been described that are capable of inducing potent T cell and antibody responses against encoded transgenes [27]. When targeting the blood-stage malaria antigen MSP1, high level antibody-mediated protection could be achieved in the *P. yoelii* mouse model of blood-stage malaria infection by using a priming immunization with a recombinant human adenovirus serotype 5 (AdHu5) vector followed by a booster immunization with the poxvirus vector modified vaccinia virus Ankara (MVA) [28]. The same regime induced effector CD8^+^ T cells that could reduce *P. yoelii* parasite burden during the preceding liver-stage infection [29]. AdHu5 and poxvirus vaccines encoding *P. falciparum* MSP1 and AMA1 have also been reported [30], [31], [32]. The advantages of using recombinant adenovirus vectors as vaccine carriers are numerous and certain serotypes, such as AdHu5, are highly immunogenic [27]. However, the host generates an immune response not only to the transgene but to the vector as well [33], [34]. AdHu5 vectors have been developed for vaccine delivery for several diseases and tested in rodents, primates and recently in humans as a vectored vaccine against HIV-1 [35] and malaria . Anti-AdHu5 immunity has been shown in pre-clinical and clinical studies to hamper the immunogenicity of recombinant AdHu5 vaccines [36], [37], [38]. Due to the need to overcome this problem, simian adenovirus vaccine vectors, such as chimpanzee ChAd63 (previously known as AdCh63), have been developed for which there is less pre-existing immunity in human populations [39], [40]. We have recently reported that this vector exhibits comparable immunogenicity to AdHu5 when recombinant for the *P. falciparum* liver-stage antigen TRAP and blood-stage antigen MSP1 [32], [41], [42]. Here we report the iterative development of human and simian adeno- and pox-viral vectored vaccines targeting two alleles of PfAMA1. These vectors were tested in mice and rabbits to assess the cellular and humoral immunogenicity of these vaccines in a prime-boost vaccination regime. PfAMA1-specific antibody induction by these viral vectors was optimized by using mammalian secretory signal sequences and inclusion of the native transmembrane domain for cell surface antigen localization. These data support the rational design of vectored vaccine transgene inserts for optimization of immune responses against multiple alleles of the same antigen.

## Results

### Antibody responses induced by AdHu5-MVA PfAMA1 (3D7) prime boost vaccination in mice and rabbits

**Figure 1:**
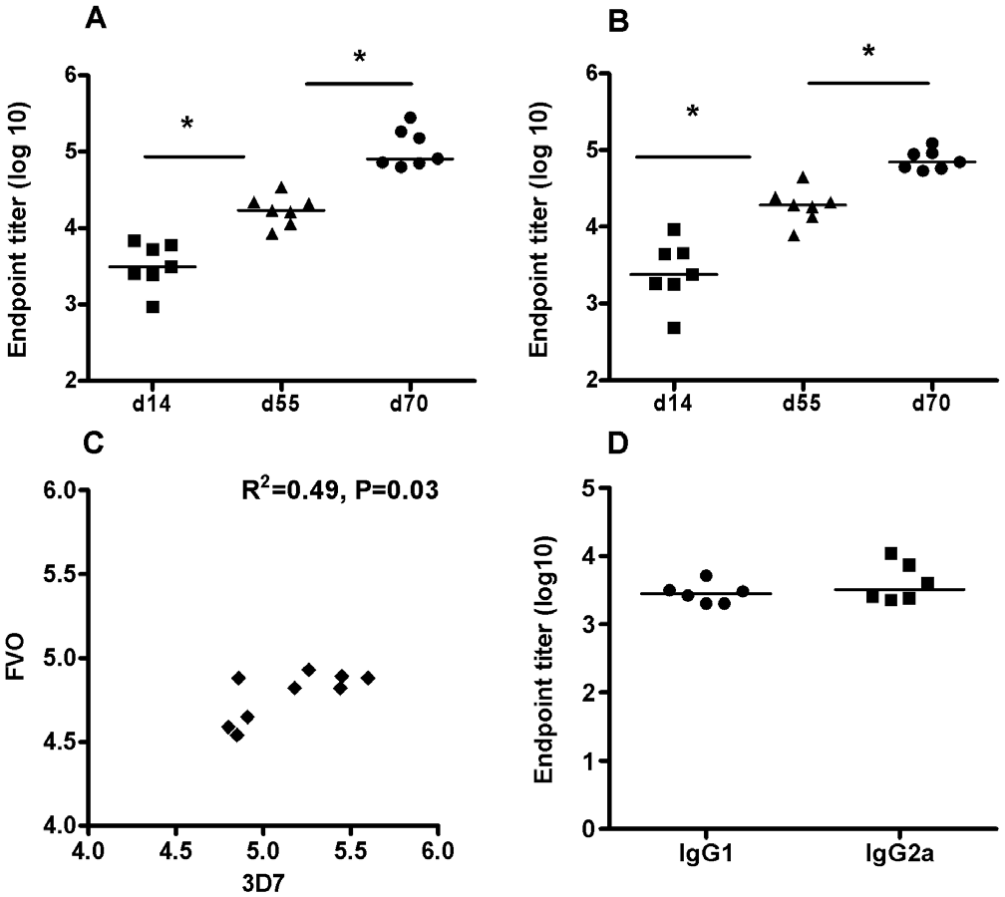
Murine antibody responses generated by AdHu5-MVA PfAMA1 (3D7) vaccination. 5–6 week old female BALB/c mice (*n* = 7/group) were immunized via the intradermal route with 5×10^10^ vp of AdHu5-PfAMA1 and boosted 8 weeks later with 1×10^7^ pfu MVA-PfAMA1. The AMA1 specific antibody response against both homologous 3D7 (A) and heterologous FVO (B) PfAMA1 recombinant protein was measured in the serum at day 14 (post prime), 55 (pre boost) and 70 (post boost). Data show the responses from individual mice and the lines represent the median. The asterisk indicates a significant difference between the different time-points as analyzed by Wilcoxon signed rank test (* *P *≤0.05). (C) The correlation between the 3D7 and FVO antibodies at day 70 was analyzed using Pearson's rank correlation (R^2^ = 0.49, *P *≤0.05). (D) IgG isotypes were assessed at day 70 in the serum against recombinant FVO PfAMA1.

PfAMA1-specific IgG antibody responses were measured by ELISA in the serum of BALB/c mice and New Zealand white rabbits after an AdHu5-MVA PfAMA1 (3D7) heterologous prime-boost vaccination regimen using an eight week interval [28]. Both vectors encoded a first generation PfAMA1 (3D7) vaccine construct (aa 25–546) including the tPA leader sequence and with mutations in potential N-linked glycosylation sites (as described in Methods). In mice the kinetics of the total IgG response was measured by ELISA against both the homologous 3D7 allele (vaccine strain) (Figure 1A) and the heterologous FVO allele (Figure 1B) to assess the induction of cross-reactive antibodies. All mice seroconverted after the AdHu5-PfAMA1 (3D7) prime (assessed at day 14) and these responses significantly increased (*P*≤0.05) by day 55 (pre-boost), in agreement with murine data for similar AdHu5 vectors encoding the *P. yoelii* and *P. falciparum* MSP1 antigens [28], [32]. The MVA boost immunization on day 56 significantly increased (*P*≤0.05) the PfAMA1-specific IgG antibody response as assessed on day 70 (two weeks post boost). Cross-reactive antibodies were measured against the heterologous FVO allele and there was a significant correlation (R^2^ = 0.49, *P* = 0.03) between the antibodies to 3D7 and FVO PfAMA1 at the peak of the response, day 70 (Figure 1C). The IgG isotypes (IgG1 and IgG2a) were also measured in the sera and there was a balanced Th1-type IgG2a and Th2-type IgG1 response in mice at the day 70 time-point (Figure 1D).

**Figure 2:**
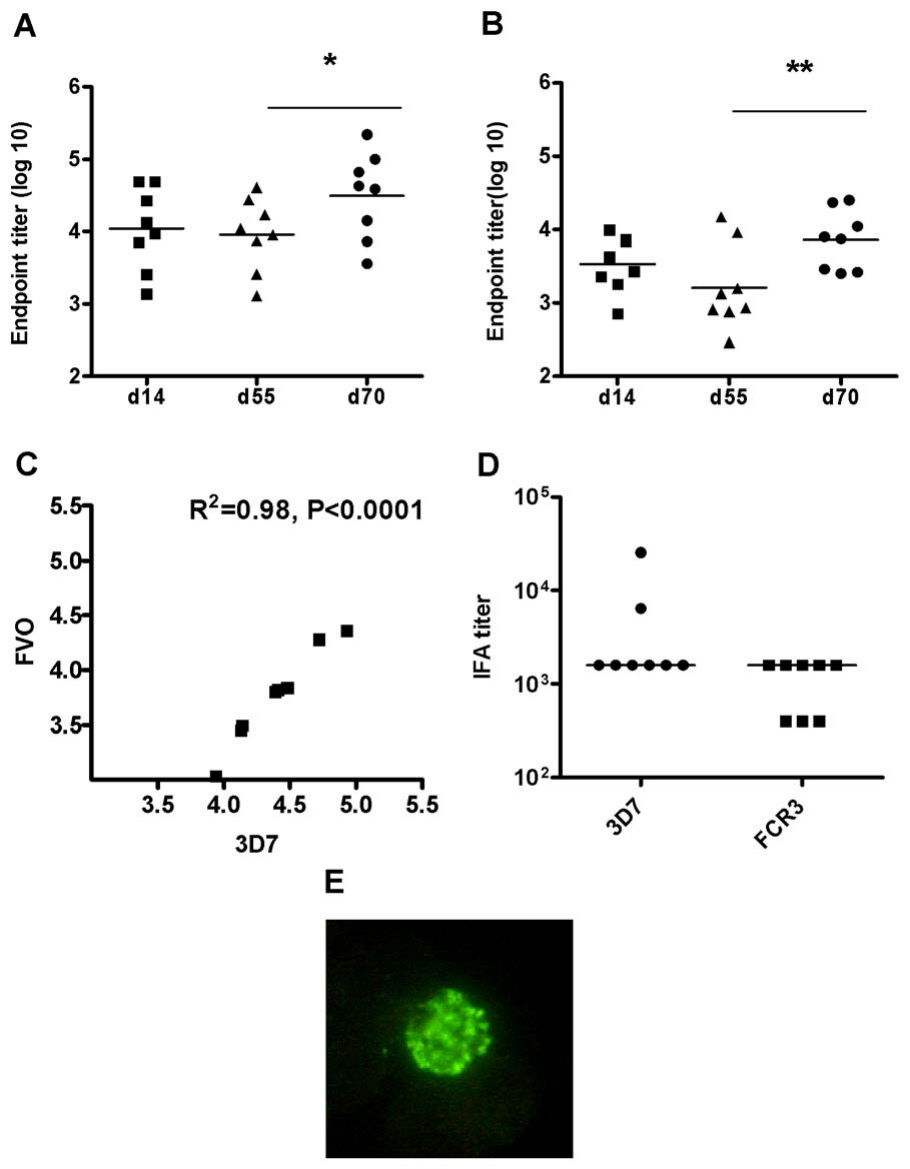
*P. falciparum* AMA1 specific total IgG responses induced by immunization in rabbits. New Zealand rabbits (*n* = 8) were immunized via the intradermal route with 5×10^10^ vp of AdHu5-PfAMA1 (3D7) and boosted 8 weeks later with 5×10^7^ pfu of MVA-PfAMA1 (3D7). The AMA1-specific antibody response against both homologous 3D7 (A) and heterologous FVO (B) PfAMA1 recombinant protein was measured in the serum at day 14 (post prime), 55 (pre boost) and 70 (post boost). Data show the responses from individual rabbits and the lines represent the median. The asterisk indicates a significant difference between the different time-points as analyzed by Wilcoxon signed rank test (* *P *≤0.05, ** P ≤0.01). (C) The correlation between the 3D7 and FVO antibodies at day 70 was analysed using Pearson's rank correlation. There was a highly significant positive correlation (R^2^ = 0.96, *P *≤0.0001). (D) IFA titers were measured using sera taken two weeks following the boost immunization. Individual IFA titers against 3D7 and FCR3 *P. falciparum* strains are shown with the median response (FCR3 AMA1 differs from FVO AMA1 by only a single amino acid substitution [18]). (E) When serum from immunized rabbits was used to label *P. falciparum* schizonts, typical AMA1-specific punctate apical staining of merozoites was seen.

The kinetic of the IgG antibody response in rabbits after the same immunization regime was different to that seen in mice. After immunization with AdHu5-PfAMA1 (3D7) all the rabbits seroconverted, but the antibody response tended to be lower at day 55 post prime in comparison to day 14 (*P*≤0.05) (Figure 2A,B), unlike in mice where there was a significant increase in the antibody response over time after the AdHu5-PfAMA1 (3D7) prime. Administration of the MVA boost on day 56 significantly increased the antibody response by day 70. Measurement of cross-reactive antibodies against FVO AMA1 showed there was a stronger correlation between the 3D7 and FVO antibodies at day 70 in rabbits (R^2^ = 0.98, *P*≤0.0001) than in mice (Figure 2C). The antibodies induced by vaccination recognized native AMA1 protein as shown by parasite immunofluorescence assay (IFA) (Figure 2D). IFA was performed using slides of *P. falciparum* schizont infected red blood cells (RBCs) which were incubated with day 70 sera from immunized rabbits. The sera bound to the *P. falciparum* schizont stages as shown by the punctate apical staining of merozoites that is typical of that seen for AMA1 (Figure 2E).

### Functional activity of antibodies generated in mice and rabbits

**Figure 3:**
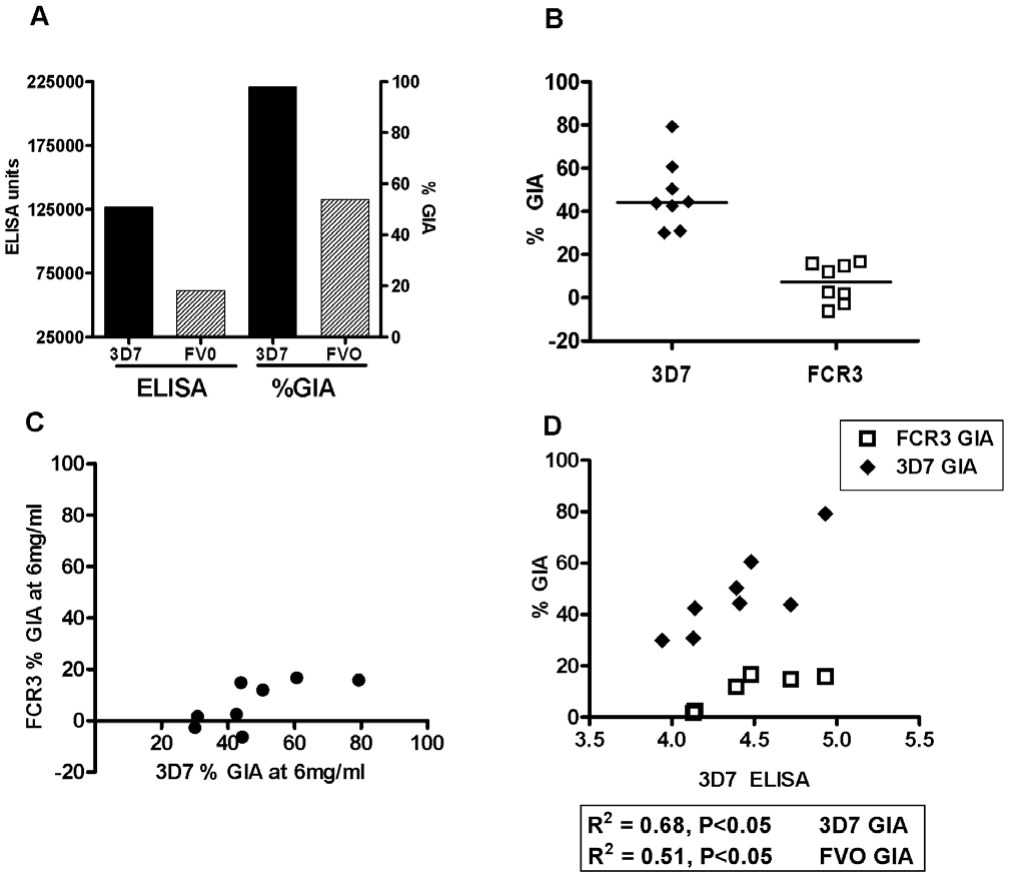
Growth inhibitory activity of sera from immunized mice and rabbits. 5–6 week old female BALB/c mice (*n* = 7) were immunized as in Figure 1. Sera were obtained two weeks post boost (day 70) by terminal bleed and the IgG was purified from the pooled sera. The GIA activity of the serum was assessed by incubating the IgG at a final concentration of 5 mg/ml with *P. falciparum* cultures. The ELISA titers and the % growth inhibition of the pooled serum are shown against both the homologous 3D7 and heterologous FVO strains of PfAMA1 (A). New Zealand white rabbits (*n* = 8) were immunized as in Figure 2. Two weeks after the final vaccination the sera were obtained. The effect of the purified IgG on parasite growth from each individual rabbit was evaluated by incubating the IgG at a concentration of 6 mg/ml with mature *P. falciparum* schizonts. The % GIA values for individual rabbits and the median are shown against both the 3D7 and FCR3 strains at 6 mg/ml IgG concentration (B). There was no correlation between the 3D7 and FCR3 GIA (C). There was a significant positive correlation between the 3D7 ELISA titers and 3D7 GIA (R^2^ = 0.68, *P ≤0.05) and with FCR3 GIA (R^2^ = 0.51, *P ≤0.05) using Pearson's rank correlation (D).

The functional activity of antibodies induced by the AdHu5-MVA PfAMA1 (3D7) vaccination regime was determined using a standardized assay of growth inhibitory activity (GIA) [43], which measures the growth inhibition of blood-stage *P. falciparum* parasites by purified IgG. Mouse sera obtained at the day 70 time-point were pooled and the GIA was determined using a final concentration of 5 mg/mL purified IgG. The samples showed a GIA of 98% against 3D7 parasites (vaccine strain), but lower levels against the heterologous FVO parasites (54%) (Figure 3A). Purified control mouse IgG shows no significant biological activity against either parasite strain in this assay [28]. The antibody response (measured using a standardized ELISA [11]) against the 3D7 allele was higher than FVO (Figure 3A).

Similar assays were performed for individual rabbit serum samples taken two weeks after the final immunization, and GIA was assessed against the homologous 3D7 and heterologous FCR3 strains using purified IgG at a final concentration of 6 mg/mL. The AMA1 sequence of FCR3 differs from that of the FVO strain by only a single amino acid [18]. In rabbits there was moderate growth inhibition against the homologous 3D7 strain (median = 44%, *n* = 8) but little or no GIA against the heterologous FCR3 strain (median = 7%, *n* = 8) (Figure 3B). This strain-specific GIA of purified rabbit IgG has been reported in numerous other studies using recombinant protein AMA1 vaccines [12], [19]. There was no correlation between 3D7 and FCR3% GIA (Figure 3C). However, as seen in other studies [32] there was a positive correlation between the 3D7 AMA1 ELISA titers and 3D7 GIA (R^2^ = 0.68, *P*≤0.05) and also with FCR3 GIA (R^2^ = 0.51, *P*≤0.05) (Figure 3D).

### Generation of modified mono-allelic PfAMA1 vaccine constructs

**Figure 4:**
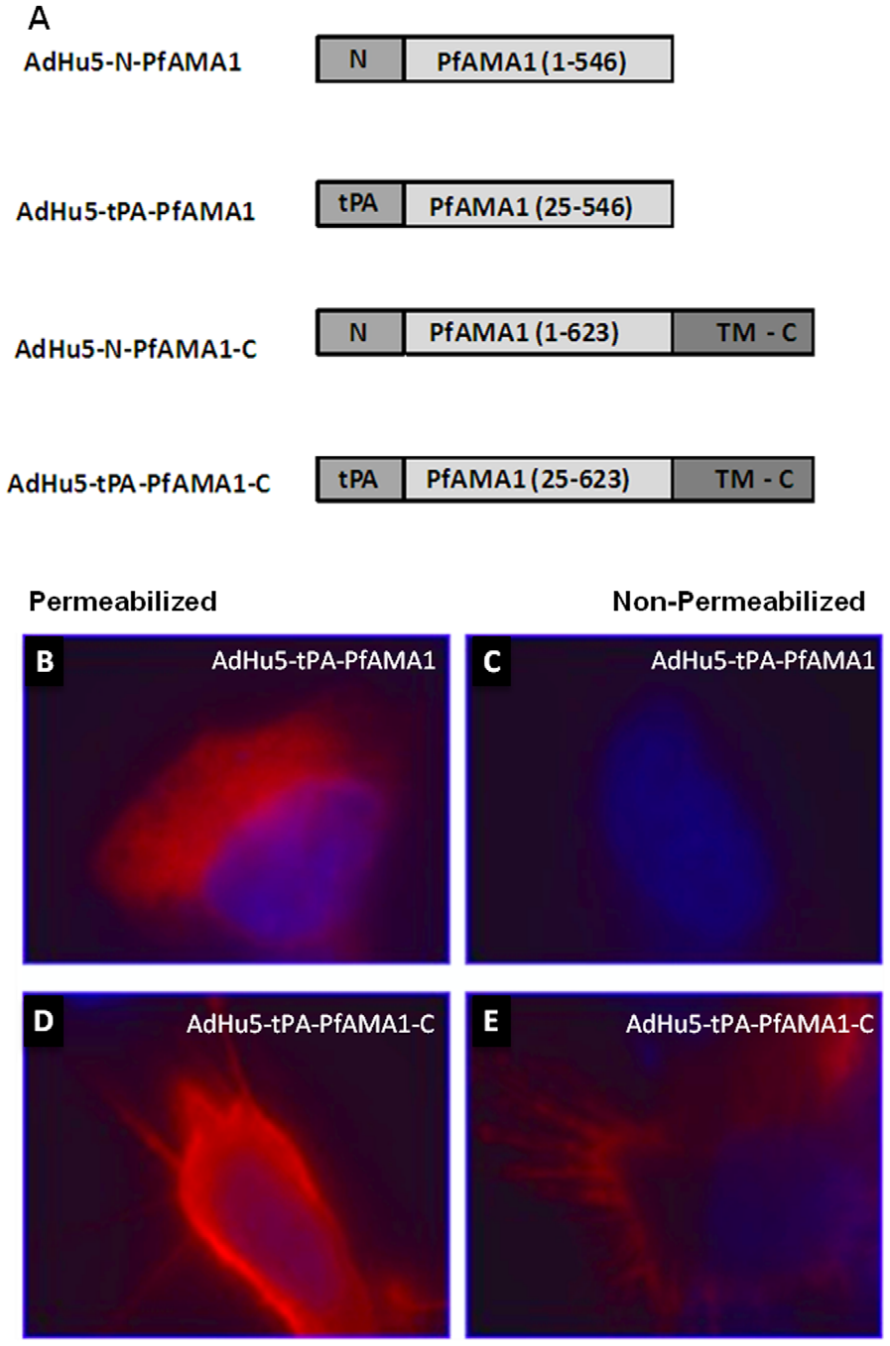
Schematic representation of the different PfAMA1 (3D7) constructs and effect of the addition of the C terminus on cellular localization of PfAMA1. (A) The figure shows the different AdHu5 vaccine inserts generated to test the effect of replacing the human tPA leader sequence with the native AMA1 N-terminal predicted signal peptide (aa 1–24) and also the effect of addition of the native transmembrane domain and C-terminal cytoplasmic domain (aa 547–623). PfAMA1 in this figure represents the ectodomain of *P. falciparum* 3D7 AMA1 (aa 25–546). Hela cells were grown on cover slips and transfected with the plasmid encoding tPA-PfAMA1 (B and C) or tPA-PfAMA1-C (D and E). After overnight incubation polyclonal serum from immunized mice was used to detect the localization of PfAMA1. Alexa-conjugated anti-mouse antibody was used as the secondary antibody and the DNA was stained with 4′, 6-diamidino-2-phenylindole (DAPI). Cell surface localization of PfAMA1, notably on the cell projections, was seen in the construct that has the C terminal domains in both the permeabilized (D) and non-permeabilized samples (E). There is no cell surface associated PfAMA1 in the construct without the C terminal domains (B and C).

These preliminary data in mice and rabbits highlighted the need to further optimize the PfAMA1 vaccine constructs in terms of inducing antibodies capable of inhibiting the growth of divergent *P. falciparum* strains. One recent study has demonstrated the ability of co-immunization with two alleles of AMA1 to focus the induced antibodies on conserved epitopes [18] and another demonstrated enhanced antibody-inducing capability of an AdHu5 vaccine expressing PfAMA1 (3D7) when localized to the cell surface in comparison to intracellular expression [30]. The first generation mono-allelic PfAMA1 vaccine described here utilized the signal peptide from the human tissue plasminogen activator (tPA) as a leader sequence in place of the native parasite N-terminal (N) PfAMA1 signal sequence. However, the transmembrane domain (TM) and the cytoplasmic tail (C) of PfAMA1 were not included in this construct. As part of the process to develop a second generation vaccine construct based on PfAMA1, the potential effects on antibody immunogenicity of the addition of the native N and/or C termini were tested. Three new recombinant AdHu5 vectors were thus generated (second generation mono-allelic PfAMA1 (3D7) vaccines) encoding the ectodomain of PfAMA1 (aa 25–546) with either the native N terminal signal peptide or tPA leader and either with or without the native C-terminal transmembrane and cytoplasmic domains (aa 547–623) (Figure 4A), in order to comparatively assess antibody immunogenicity.

### Effect of the addition of the C-terminus on cellular localization of PfAMA1

In order to confirm differences in the subcellular localization of the PfAMA1 antigen following inclusion of the C-terminus in the vaccine construct, an *in vitro* immunofluorescence assay was performed. Transfection of HeLa cells with plasmid construct tPA-PfAMA1-C containing the AMA1 C-terminus showed a cell surface localization of PfAMA1 in both the permeabilized and non-permeabilized cells (Figure 4D,E). In contrast, when using the construct tPA-PfAMA1 that expressed the construct without the C-terminal membrane anchor, PfAMA1 expression was only detectable in the permeabilized cells, presumably localized in the endoplasmic reticulum (Figure 4B,C). These data confirmed that the addition of the C-terminus led to the cell surface expression of PfAMA1.

### Effect of replacing the native parasite signal peptide with tPA as a leader sequence

**Figure 5:**
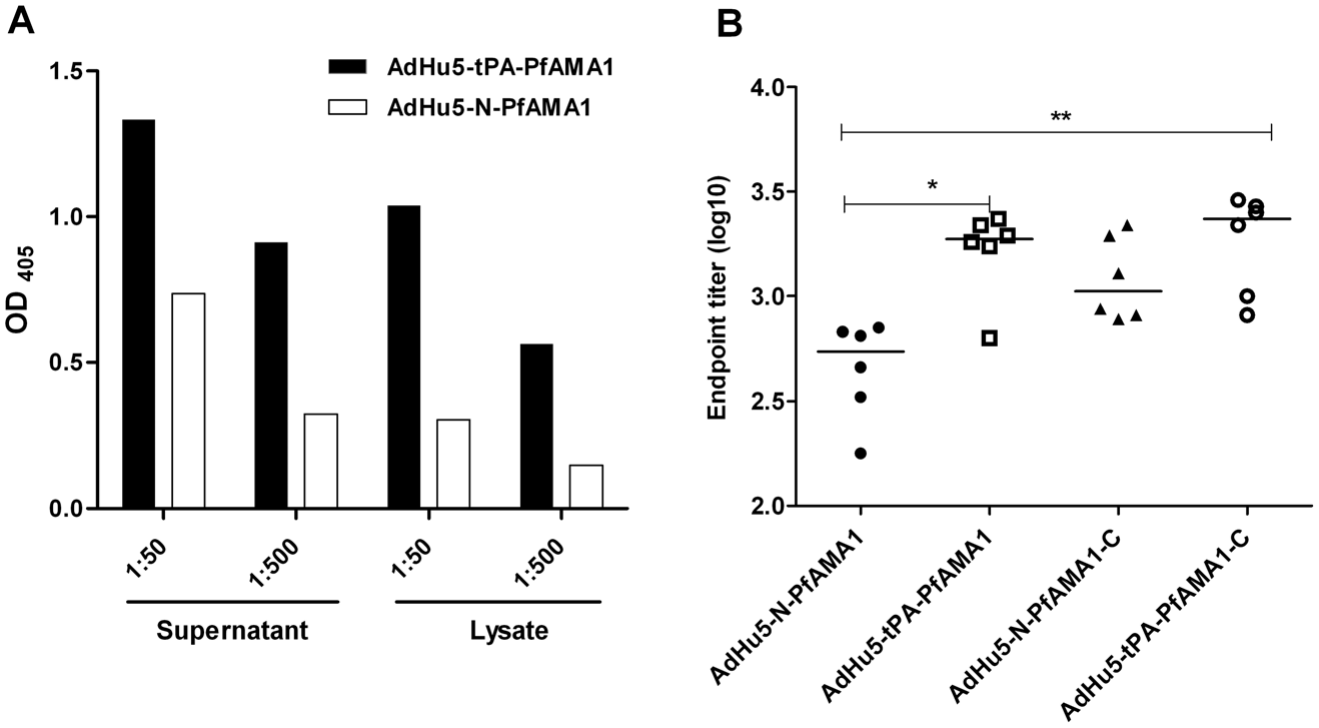
Use of a mammalian secretory signal sequence enhances antigen expression and antibody immunogenicity of an AdHu5 vectored PfAMA1 vaccine. (A) A549 cells were infected either with the same number of infectious units of AdHu5-tPA-PfAMA1 or AdHu5-N-PfAMA1. The quantity of PfAMA1 protein was assessed in the supernatant and lysate by a PfAMA1 capture ELISA. The figure shows the OD_405_ values for both the supernatant and the lysate for the two constructs. (B) Four groups of 5–6 week old female BALB/c mice were immunized with one of the four constructs i) AdHu5-tPA-PfAMA1, ii) AdHu5-tPA-PfAMA1-C, iii) AdHu5-N-PfAMA1, or iv) AdHu5-N-PfAMA1-C at a dose of 1×10^10^ vp. The total IgG response was measured by ELISA in the serum 12 days after immunization against *P. falciparum* 3D7 recombinant protein. The median and endpoint titers (log 10) for individual mice are shown. The asterisk indicates a significant difference in endpoint titer between the groups by ANOVA (* *P *≤0.05, ** *P *≤0.01).

The immunoflourescence assay does not quantify the effect of replacing the N-terminal parasite signal sequence with tPA, so a capture ELISA was performed to quantify the amount of PfAMA1 protein secreted/expressed from the two vectors: i) AdHu5-tPA-PfAMA1 and ii) AdHu5-N-PfAMA1. In agreement with the hypothesis that the mammalian leader sequence could enhance transgene secretion and/or protein expression, there was consistently more PfAMA1 protein in both the supernatant and the lysate of the cells infected with the vector containing the tPA signal peptide as compared to those infected with the vector containing the native parasite AMA1 signal peptide (Figure 5A).

### Comparative immunogenicity of modified PfAMA1 transgene inserts

Four groups of BALB/c mice were subsequently immunized with the four different AdHu5 constructs: i) AdHu5-tPA-PfAMA1, ii) AdHu5-tPA-PfAMA1-C, iii) AdHu5-N-PfAMA1 and iv) AdHu5-N-PfAMA1-C. The total IgG response to the ectodomain of PfAMA1 was measured by ELISA in the serum 12 days post vaccination against recombinant PfAMA1 (3D7) protein (Figure 5B). Inclusion of the human tPA leader sequence in the vaccine led to significantly higher (*P*≤0.05) antibody titers than when the native parasite N-terminus was used, presumably due to increased efficiency of antigen secretion and/or expression (Figure 5A). Interestingly, the antibody response was maintained when the native C-terminus was included in the vaccine construct in conjunction with the tPA leader, and in fact responses tended to be stronger when the C-terminus was used in conjunction with the native N-terminal signal sequence. However, the addition of the C-terminus did not further enhance the antibody response when the tPA leader sequence was used in place of the native N-terminus. These data indicate that although cell surface localization may in some cases enhance the antibody immunogenicity of AdHu5 recombinants, the efficiency of antigen secretion and/or level of expression may be of greater importance.

### Generation and immunogenicity of a bi-allelic PfAMA1 construct

A bi-allelic PfAMA1 vaccine construct was designed in an attempt to address the issue of antigenic polymorphism in PfAMA1. The 3D7 and FVO alleles utilized in other candidate protein vaccines [17] were included and are two of the most divergent in terms of amino acid (aa) polymorphism differing by 24 aa [20]. The construct contained the ectodomains of the two alleles fused in tandem and linked by a glycine-proline linker, as well as the tPA leader sequence (at the N-terminus) and the TM region and C-terminus of the FVO allele [44]. For pre-clinical studies this construct was cloned into MVA (referred to as MVA-bi-allelic), AdHu5 (AdHu5-bi-allelic) and the chimpanzee adenovirus ChAd63 (ChAd63-bi-allelic). This simian vector has also been used in the development of other *P. falciparum* candidate malaria vaccines encoding the ME-TRAP [41] and MSP1 [32], [42] antigens, and was used in order to circumvent issues surrounding pre-existing anti-vector immunity to AdHu5 in humans.

**Figure 6:**
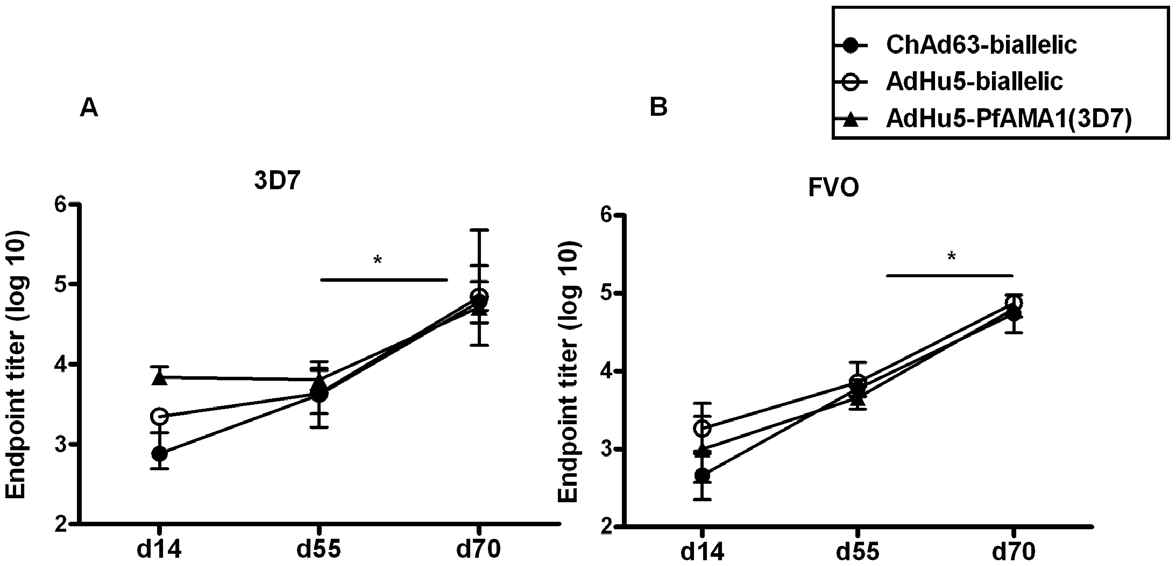
Antibody responses in mice induced by the mono-allelic and bi-allelic PfAMA1 vaccine constructs. 6 week old female BALB/c mice (*n* = 6) were primed with 1×10^9^ vp of each of the adenovirus constructs i) ChAd63-biallelic, ii) AdHu5-biallelic and iii) AdHu5-PfAMA1 (3D7). Groups i) and ii) were boosted at day 56 with 1×10^7^ pfu of MVA-biallelic and group iii) was boosted with the mono-allelic MVA-PfAMA1 (3D7). The total IgG response was measured by ELISA in the serum at day 14, 55 and 70 against both the 3D7 (A) and FVO (B) alleles. The median and the range of the endpoint titer (log 10) are shown in the figure. There were significant differences between the ELISA titres at day 14 by one-way ANOVA: 3D7 PfAMA1 ChAd63-biallelic versus AdHu5-monoallelic *P *<0.001; FVO PfAMA1 ChAd63-biallelic versus AdHu5-biallelic *P *<0.05. The MVA boost significantly increased IgG titres against both alleles in all groups (* *P *<0.05 by Wilcoxon signed rank test).

Mice were primed with either i) ChAd63-bi-allelic, ii) AdHu5-bi-allelic or iii) the mono-allelic AdHu5-PfAMA1 (3D7) vectors. Groups i) and ii) were boosted with MVA-bi-allelic and group iii) was boosted with the mono-allelic MVA-PfAMA1 (3D7) vaccine. Total IgG was measured in the serum on days 14, 55 and 70 by ELISA against recombinant 3D7 and FVO PfAMA1. AMA1 specific IgG responses were detected in all the groups to both 3D7 (Figure 6A) and FVO (Figure 6B) recombinant proteins. Although minor, but statistically significant differences were noted between groups by one-way ANOVA analysis at day 14, by day 55 these differences in IgG antibody responses had equalized between the different groups, suggesting that after priming with ChAd63 rather than AdHu5, the antibodies take slightly longer to reach maximal levels. The MVA immunization significantly increased the antibody response against both alleles in all the groups to similar levels by day 70 (*P*≤0.05). Thus, in agreement with data for the MSP1 antigen [32], the ChAd63 and AdHu5 vectors expressing the bi-allelic PfAMA1 construct primed similar IgG responses, which remained equivalent after a heterologous MVA boost vaccination. However, the immunogenicity of the mono-allelic and bi-allelic AMA1 vaccine constructs was indistinguishable based on the absolute IgG titers.

### Comparison of antibody responses and serum GIA generated in rabbits after vaccination with mono-allelic or bi-allelic PfAMA1 vaccines

**Figure 7:**
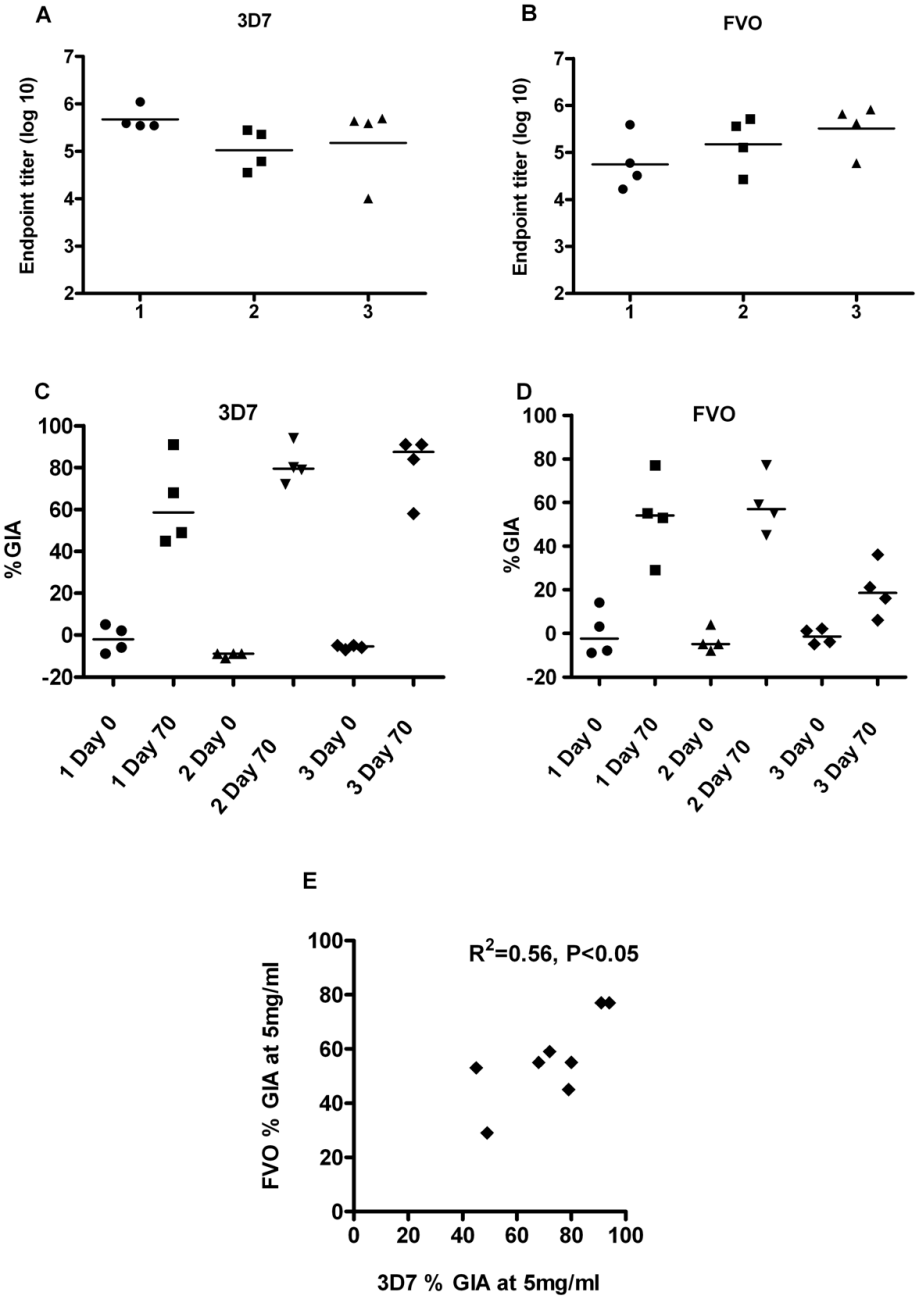
Comparison of antibody and growth inhibitory activity of rabbit IgG after vaccination with mono-allelic or bi-allelic PfAMA1 vaccines. New Zealand rabbits (*n* = 4) were primed with 1×10^10^ vp 1) ChAd63-biallelic, 2) AdHu5-biallelic or 3) AdHu5-PfAMA1 (3D7). Groups 1) and 2) were boosted with 1×10^7^ pfu of the bi-allelic MVA construct and group 3) was boosted with the mono-allelic MVA-PfAMA1 (3D7). The AMA1 specific antibody response against both homologous 3D7 (A) and FVO (B) PfAMA1 recombinant protein was measured in the serum two weeks after the final immunization (day 70). There was no significant difference in the antibody responses between the groups against the 3D7 or FVO alleles. The % GIA values for individual rabbits and the median are shown against both the 3D7 (C) and FVO (D) strains at 5 mg/ml IgG concentration. There was a significant positive correlation between the 3D7 and FVO GIA (R^2^ = 0.56, *P ≤0.05) (E).

New Zealand white rabbits (*n* = 4) were primed with 1) ChAd63-bi-allelic, 2) AdHu5-bi-allelic or 3) the mono-allelic AdHu5-PfAMA1 (3D7) vaccines. Groups 1) and 2) were boosted with MVA-bi-allelic and group 3) was boosted with the mono-allelic MVA-PfAMA1 (3D7) vaccine. Antibody levels were measured two weeks after the final vaccination against 3D7 and FVO PfAMA1 (Figure 7A,B). As seen in mice, there was no significant difference in the antibody response generated after the three vaccination regimes either against 3D7 or FVO PfAMA1. Cross-reactive antibodies were measured against the FVO recombinant protein after immunization with the AdHu5-MVA PfAMA1 (3D7) vaccination regime as seen previously (Figure 2B). However, when antibody functionality was assessed using the *in vitro* assay of GIA, the mono-allelic vaccine once again induced strain-specific activity: 87.5% GIA against the 3D7 parasite but only 18.5% GIA against FVO (Figure 7C,D), in agreement with the previous study (Figure 3B). However the ChAd63-MVA bi-allelic and AdHu5_MVA bi-allelic vaccination regimes induced antibodies that showed comparable GIA against both the 3D7 (58.5% and 79.5% respectively) and FVO (54% and 57% respectively) parasites. GIA against 3D7 and FVO parasite strains following bi-allelic immunization also correlated significantly (Figure 7E). These data confirm that even though mono- and bi-allelic PfAMA1 vaccine constructs induce cross-reactive IgG that are indistinguishable by ELISA assay, the IgG induced by the bi-allelic vaccines was capable of reducing the growth of both parasite strains encoded by the vaccine *in vitro*.

### T cell immunogenicity and multi-functionality of the mono-allelic and bi-allelic PfAMA1 vaccines in mice

**Figure 8:**
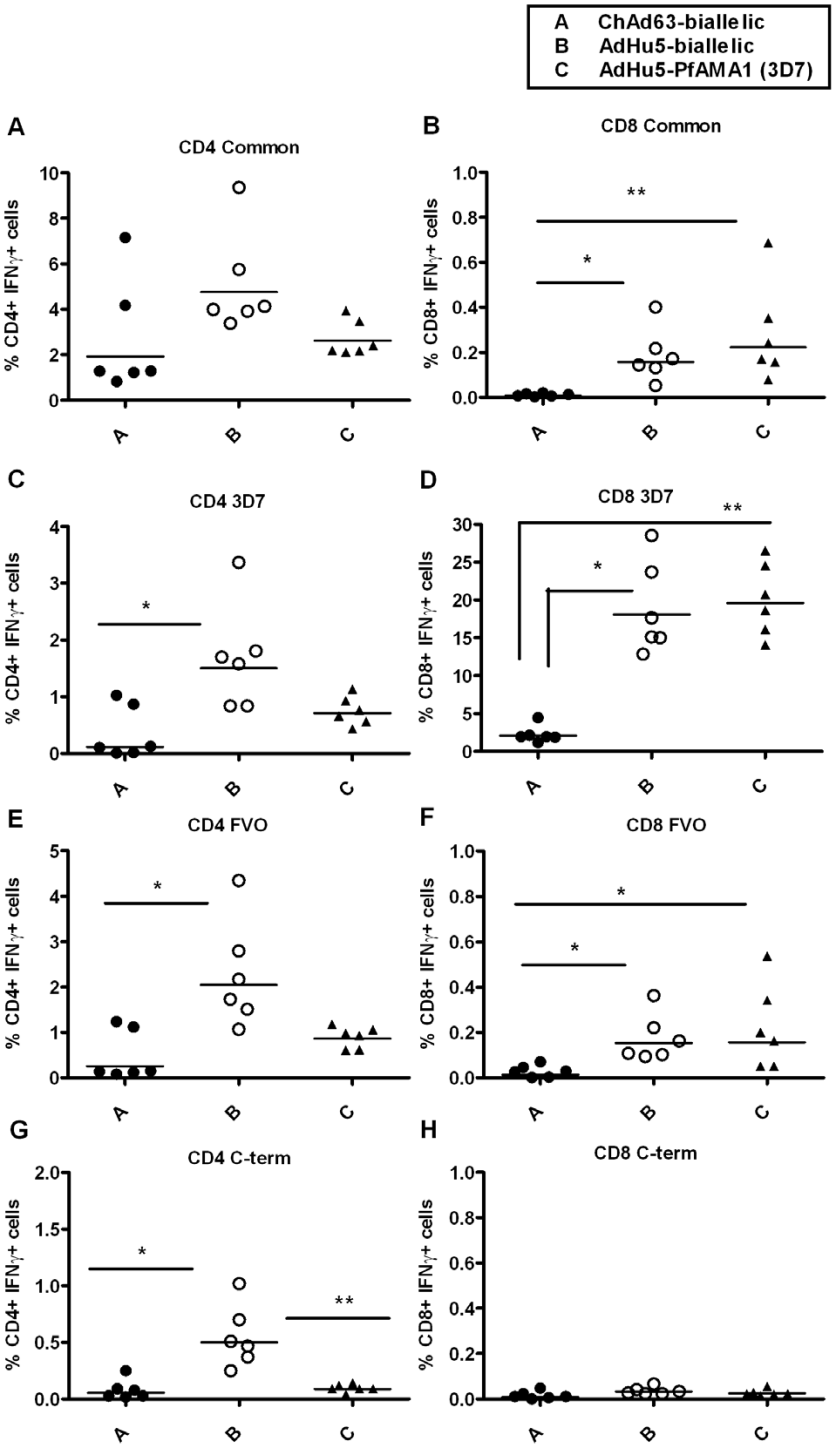
T cell responses induced in mice by vaccination with the mono-allelic and bi-allelic constructs. 5–6 week old female BALB/c mice (*n* = 6) were immunized as in Figure 6. Splenocytes were isolated two weeks after the final immunization (day 70) and stimulated with four peptide pools (Common, 3D7, FVO and C-terminus) or no peptide (Unstim) for 5 hours. After this time the cells were surface stained for CD4 and CD8 and then intracellularly stained for IFN-γ, TNFα and IL-2 and analysed by flow cytometry for % of responding cells per total CD4^+^ or CD8^+^ T cell subset. % CD4^+^ and CD8^+^ T cells positive for IFN-γ, TNF-α or IL-2 following the different vaccination regime is shown (8A-H). Individual data points and the median are shown. The asterisk indicates a significant difference between the groups by ANOVA (* *P *≤0.05, ** *P *≤0.01).

T cell responses were also analyzed in BALB/c mice by ICS following re-stimulation of splenocytes with one of four pools of overlapping peptides (15 mers, overlapping by 10 aa) corresponding to the 3D7 and FVO coding sequence in the bi-allelic vaccine [44]. Pool 1 contained 64 peptides common to both alleles of PfAMA1. Pools 2 and 3 (47 peptides in each) contained allele-specific peptides unique to 3D7 and FVO respectively (each peptide pair differ by 1 or 2 aa). Pool 4 had only 12 peptides corresponding to the C-terminus of the FVO allele. Splenocytes were isolated from the mice described in Figure 6 on day 70 and re-stimulated with the four peptide pools or no peptide control (Unstim) for 5 hours. After this, cells were surface stained for CD4 and CD8 and then intracellularly stained for IFN-γ, TNFα and IL-2, before analysis by flow cytometry for % of responding cells per total CD4^+^ or CD8^+^ T cell subset (Figure 8A–H).

CD8^+^ T cell responses were detected to the common, 3D7 and FVO peptide pools. In all the three pools the ChAd63-MVA bi-allelic PfAMA1 regime induced a significantly weaker CD8^+^ T cell response than the equivalent AdHu5-MVA regime (comparing groups A and B, *P*≤0.01). Interestingly, there were cross-reactive and comparable CD8^+^ T cell responses detected to the FVO pool when mice were immunized with the AdHu5-MVA AMA1 (3D7) mono-allelic regime (comparing groups B and C), indicating the amino acid substitutions between the two allelic forms were either not present in the epitope(s) recognized, or did not affect class I MHC presentation or TCR recognition. The dominant CD8^+^ T cell response in BALB/c mice was to an epitope(s) in the 3D7 pool. There was no response detected to the C-terminal region.

CD4^+^ T cell responses were detected to all four peptide pools, although the overall total magnitude of the response was lower than the total CD8^+^ T cell response. Consistent with the CD8^+^ T cell data, the ChAd63-MVA bi-allelic PfAMA1 regime induced an overall weaker CD4^+^ T cell response in comparison to the equivalent AdHu5-MVA regime (comparing groups A and B, *P*≤0.05 for 3D7 and *P*≤0.01 for FVO and C-term). The bi-allelic AdHu5 vaccine construct tended to induce stronger responses against the common, 3D7 and FVO pools in comparison to the equivalent AdHu5 mono-allelic regime (comparing groups B and C) but this did not reach statistical significance. Also as expected, there was no response detected to the C-terminus when mice were immunised with the mono-allelic 3D7 construct (which does not contain the C-terminus), however the AdHu5 bi-allelic construct which does contain this sequence induced detectable CD4^+^ T cell responses to this peptide pool.

**Figure 9:**
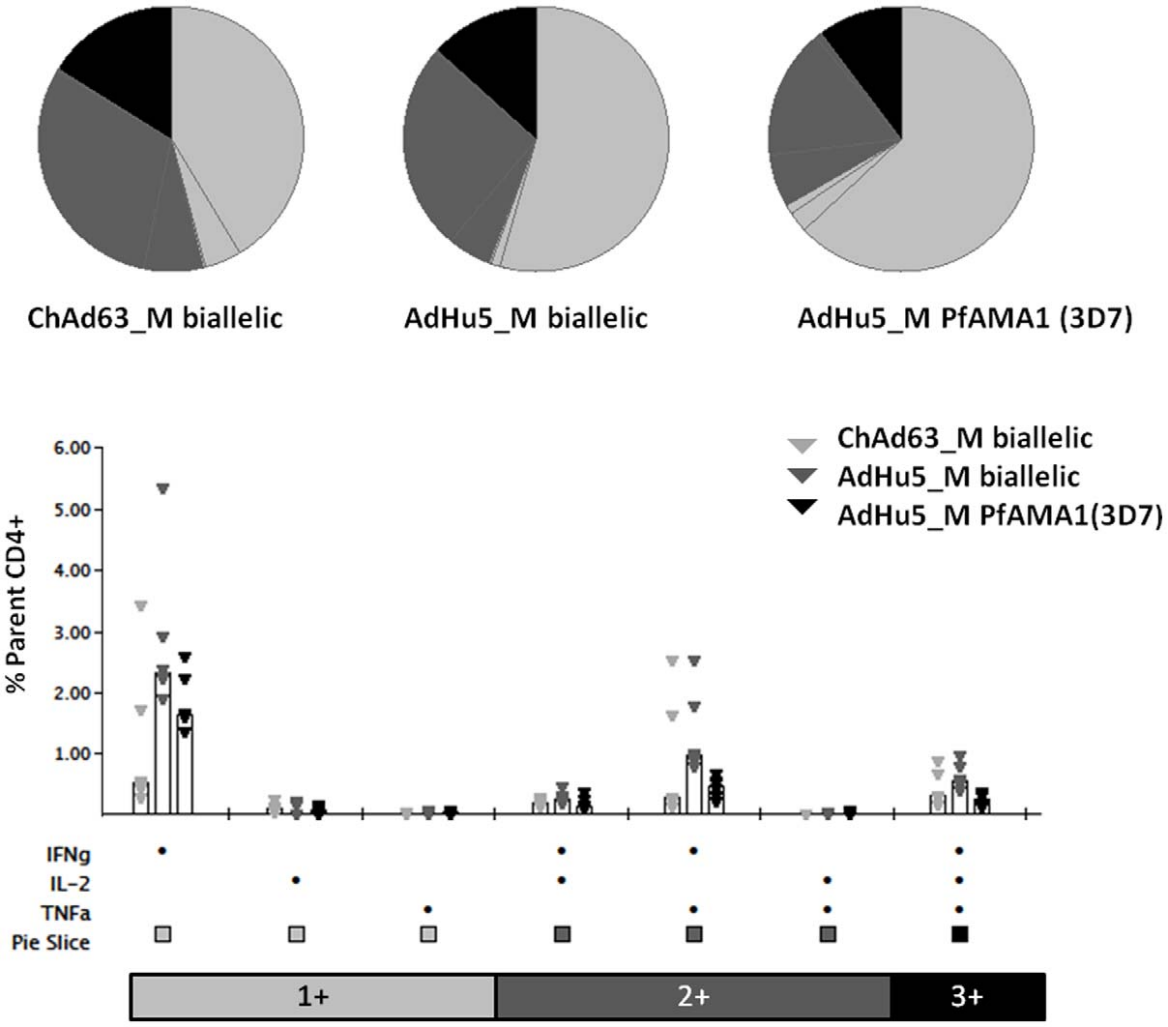
Multi-functionality of CD4^+^ T cell response induced by vaccination. The multi-functional composition of the CD4^+^ T cell response (measured in Figure 8) is shown. The pie charts summarize the fractions of AMA1-specific CD4^+^ cells that are positive for a given number of functions (IFN-γ, TNF-α and IL-2). Individual data points and median percentage of the parent CD4^+^ T cell response (open bars) are shown for each of the functional populations indicated on the x-axis.

Cross-reactive CD4^+^ T cell responses were again detected to the FVO pool of peptides when mice were immunized with the mono-allelic 3D7 vaccine, possibly for similar reasons as for the CD8^+^ T cell responses. The induction of CD4^+^ T cells against common epitopes could be relevant to the issue of AMA1 polymorphism if these epitopes are important for protection, as indicated by studies of AMA1 in the *P. chabaudi* rodent malaria model [5], [45]. The multi-functionality of the CD4^+^ T cell response to the common pool was also assessed, given CD4^+^ T cells producing IFN-γ, TNFα and IL-2 simultaneously have been shown to correlate with protection against *Leishmania major* in mice [46]. All three vaccination regimes induced multi-functional CD4^+^ T cells, and there were no significant differences in the quantity or quality of multi-functional cells among the different vaccination regimes tested (Figure 9).

## Discussion

This paper describes the development, immunogenicity and *in vitro* efficacy of a viral vectored vaccination strategy targeting one of the leading blood-stage malaria vaccine candidate antigens PfAMA1. This adenovirus-MVA prime-boost regime induces antibody responses in mice and rabbits, and these antibodies inhibit growth of *P. falciparum* parasite strains *in vitro* according to the alleles of PfAMA1 encoded by the recombinant viruses. The same vaccination strategy encoding another blood-stage vaccine candidate antigen (*P. falciparum* MSP1) has been shown to be similarly effective [32]. In addition to antibodies, this regimen also induces substantial antigen-specific CD8^+^ and CD4^+^ T cells in mice. The viral vectored vaccine platform is now offering a safe and reliably immunogenic alternative to recombinant protein-in-adjuvant vaccines not only for malaria vaccine development [47], but also for a wider range of difficult infectious diseases [27].

The first generation AdHu5-MVA mono-allelic AMA1 (3D7) vaccines induced cross-reactive antibodies to the heterologous FVO allele of AMA1 both in mice and rabbits as measured by ELISA, however there was a better correlation in rabbits than in mice between the 3D7 and FVO antibodies at the peak day 70 time-point. This has been seen before with a *P. falciparum* MSP1 based vaccine [32], suggesting there are differences in antibody cross-reactivity and B cell epitopes recognized in different mammalian species. These observations may be reflected in differing concentrations of inter-species IgG required to elicit equivalent functional activity *in vitro* – an observation now reported for both blood-stage antigens MSP1 and AMA1 [19], and also the transmission-blocking vaccine candidate antigen Pfs25 [48]. In another study, IgG isolated from rabbits immunized with a reduced AMA1 ectodomain were reported to not have any growth inhibitory activity *in vitro* [49]. Importantly, the first generation vaccines described here induced antibodies capable of recognizing antigen in its native conformation in schizont-infected erythrocytes, an observation also reflected in the measurement of functional GIA. In this study in mice there was 94% GIA against the homologous 3D7 strain but only 54% against the heterologous strain. Similarly in rabbits there was a median GIA of 44% against 3D7 but there was no cross-reactive GIA against the FVO or FCR3 strains of *P. falciparum in vitro* afforded by purified IgG after immunization with the mono-allelic PfAMA1 (3D7) vaccine. Purified IgG from immunized mice thus shows better cross-strain *in vitro* inhibition than IgG from rabbits, though conversely in rabbits the correlation between 3D7 and FVO antibodies as measured by ELISA is stronger. These data are not in agreement with a report where the pattern of strain specificity was found to be similar in mice and rabbits [19], but do agree with other AMA1 vaccine studies, whereby the cross-reactivity of antibodies seen by ELISA was not reflected in functional GIA assays [17], [49], [50], [51]. This study also confirms observations made by others that antibodies measured by ELISA against heterologous alleles of AMA1 do not have equal effect on parasite growth *in vitro* [49]. However, reports describe a clear sigmoidal relationship between *in vitro* GIA and ELISA titer for homologous alleles of both AMA1 and MSP1 [11].

The assay of GIA remains the gold-standard for pre-clinical screening of blood-stage malaria candidate vaccines. However, a recent Phase IIa clinical trial with a recombinant AMA1 protein vaccine failed to induce significant clinical efficacy or any delay in time to clinical diagnosis even though IgG purified from the vaccinated serum showed very high levels of GIA *in vitro* [52]. More recent epidemiological studies in the field have shown a weak contribution of GIA towards reduced malaria risk [53], and other studies have shown conflicting results [54], [55]. An alternative *in vitro* assay, measuring antibody-dependent respiratory burst (ADRB) activity from neutrophils has recently been associated with clinical protection in a study from Senegal [56], and may provide a complementary assay for screening vaccine-induced IgG functional activity.

In the second generation 3D7 PfAMA1 vaccines, the substitution of the native parasite AMA1 leader sequence with the mammalian tPA leader was shown to lead to significantly improved antibody responses in mice immunized with the recombinant AdHu5 vector. The tPA signal sequence is a specific targeting signal that targets the expressed antigen to the endoplasmic reticulum (ER) [57] and influenza virus nucleoprotein when fused to tPA was shown to be expressed at higher levels and was found to be in a partially secreted form in *vitro* [58]. When fused to the tPA signal sequence, viral envelope protein E of Japanese encephalitis virus was expressed at higher levels in transfected cells than the wild-type construct and was mainly localized in the cytosolic and membrane fraction. These tPA fusion constructs elicited stronger humoral and cellular response and better protection against viral challenge in animals [59]. The increased antibody response seen here with this construct can thus be due to better secretion or enhanced PfAMA1 expression due to the inclusion of the tPA leader sequence as demonstrated by the capture ELISA assay. Either or both of these mechanisms could account for the increased immunogenicity of the AMA1 vaccine constructs in which the native signal peptide was replaced with tPA. The construct that included the PfAMA1 C-terminal domains (transmembrane and cytoplasmic) allowed cell surface localization, but this was found to improve humoral immunogenicity only in combination with the native signal peptide, and did not significantly further improve the tPA containing construct. In agreement with these data, it has been previously reported that antigens expressed at the cell surface were better than intracellular antigens at inducing antibody responses [30].

Polymorphism in AMA1 has been one of the main hurdles in vaccine development targeting this antigen. AMA1 polymorphisms are under balancing selection, especially within domains I and II that are the most polymorphic. It has been speculated that these polymorphisms are maintained due to selective pressure by the host immune system [60], [61], [62], [63]. In an attempt to overcome the allele-specific functionality of the IgG induced by the first generation vaccines, bi-allelic PfAMA1 vectored vaccines were developed that also incorporated the tPA leader and C-terminal region of one allele of PfAMA1. Multi-allele AMA1 protein vaccines have been developed by several groups. Approaches have included a 50∶50 mixture of recombinant 3D7 and FVO AMA1 antigen formulated in Alhydrogel ± CpG adjuvant (AMA1-C1) [64], as well as a diversity covering (DiCo) approach that covers 97% of the amino acid variability in PfAMA1 [21]. These multi-allelic vaccines show better cross-reactivity in terms of GIA than mono-allelic vaccines, and interestingly in one study have been shown to focus antibody responses on conserved B cell epitopes [18]. Data on human antibody responses to PfAMA1 show that affinity purified antibodies from individuals living in malaria endemic areas can inhibit *P. falciparum* growth *in vitro* [49], and Cortes *et al.* have also examined the allele specificities of plasma samples of adults from Papua New Guinea and found that antibodies against conserved regions of the molecule were much more common than allele-specific antibodies [14]. In this study in mice, the AdHu5-MVA and ChAd63-MVA bi-allelic PfAMA1 vaccination regimes induced antibodies that bound both the 3D7 and FVO recombinant proteins by ELISA. Although slightly weaker responses were observed at the day 14 time-point when using ChAd63, these responses equalized by day 55 and were boosted comparably by MVA. These antibody data are in agreement with those for similar vectors encoding *P. falciparum* MSP1 [32], and confirm that, at least in mice, ChAd63 can be used to replace AdHu5 in this adenovirus-MVA prime-boost vaccination regime without compromising the humoral immunogenicity of the transgene product. Since seropositivity against AdHu5 is too prevalent to permit its widespread deployment in humans, these data support its replacement with ChAd63 as a vaccine vector.

Notably in these mice, overall IgG titers were comparable whether using mono- or bi-allelic vaccines. Rabbits were thus immunized with both the vaccine constructs in order to purify IgG and assay for functional GIA against both the 3D7 and FVO strains of PfAMA1 to provide a comparison of the efficacy of the two vaccine constructs. Encouragingly the bi-allelic vaccines induced antibodies in rabbits that showed significant GIA against both the 3D7 and FVO strains unlike the mono-allelic vaccine. These data have also been confirmed in a recent macaque study, showing the ChAd63-MVA bi-allelic PfAMA1 regime induces significant GIA against both the 3D7 and FCR3 strains [44]. However, it has also been reported that the allele specificity of antibodies to PfAMA1 depends on the species immunized [19]. Interestingly, unlike for mice and rabbits discussed earlier, immunization of rhesus monkeys with recombinant PfAMA1 showed a completely different strain specificity pattern in terms of GIA. Even when monkeys were immunized with single-allele PfAMA1 vaccines, the elicited antibodies could bind indistinguishably to both homologous and heterologous PfAMA1 proteins on ELISA plates, and the antibodies showed cross-reactive biological activities, as judged by GIA and antigen reversal GIA assays [19]. Thus despite the evidence in our recent macaque study, these rabbit data confirm the value, at least in this species, of developing multi-allelic PfAMA1 vectored vaccines.

Splenic T cell responses in mice to both common and unique pools of peptides were also measured here by flow cytometry, and CD4^+^ T cells to the common pool displayed a multi-functional phenotype in terms of IFN-γ TNFα and IL-2 production. This is encouraging because if CD4^+^ or CD8^+^ T cells recognizing common epitopes in humans are important for protection, then they could help to confer efficacy against heterologous strains. In agreement with this hypothesis, there have been suggestions based on analysis of sequence polymorphism frequencies that the selection pressure on AMA1 domain III is mediated by T-cell immunity [13]. In another recent study, pluripotent effector memory T cells against infected erythrocytes have been associated with protection in humans after a vaccination with sporozoites and treatment with choloroquine [8]. In the *P. chabaudi* model, in agreement with another study [5], we have shown a protective role of vaccine induced CD4^+^ T cells against blood-stage infection by *in vivo* depletion and adoptive transfer studies [Biswas *et al.*, submitted]. We have also shown that MSP1-specific CD8^+^ T cells can reduce the liver-stage parasite burden in the *P. yoelii* model [29], and a similar mechanism may be relevant to AMA1 – an antigen also expressed by sporozoites [65] and most likely also late in the liver-stage of infection.

Although we have shown here that the ChAd63 vector induced comparable antibody immunogenicity to AdHu5, there were some differences in relation to other studies using this vector for the pre-erythrocytic ME-TRAP antigen [41] and the blood-stage antigen MSP1 in mice [32]. Unlike for ME-TRAP and MSP1, the AMA1-specific CD4^+^ and CD8^+^ T cell responses were lower following ChAd63_MVA immunization in comparison to those induced by AdHu5_MVA expressing the same vaccine construct. It is possible these differences may be antigen- and/or insert-specific and, in agreement with these murine data, weak AMA1-specific T cell responses were also induced in rhesus macaques following immunization with this vector, although they boosted to high levels following MVA [44].

Here we have described the rational design of bi-allelic PfAMA1 vectored vaccines that induce functional antibodies against two parasite strains. The addition of the mammalian tPA leader sequence to the adenoviral vectored vaccine insert significantly increases the antibody response, and this adenovirus-MVA prime-boost vaccination regime also induces T cell responses to conserved epitopes. The responses to conserved B and T cell epitopes following bi-allelic AMA1 immunization may improve the strain-transcending efficacy of PfAMA1 vaccines in humans. Overall, these data support the rational design of vectored vaccine transgene inserts for optimization of immune responses against multiple alleles of the same antigen, as well as the continued development of these vectors towards phase I/IIa clinical testing.

## Materials and Methods

### Generation of viral vectors expressing AMA1

The first generation mono-allelic AMA1 construct encoded the ectodomain of *P. falciparum* 3D7 AMA1 (amino acid 25–546), and included a number of amino acid substitutions to remove potential sites of N-linked glycosylation and was codon optimized for expression in humans (GeneArt, Regensburg, Germany), as described elsewhere [17]. The native predicted N-terminal signal sequence (N), transmembrane domain (TM) and the C-terminal cytoplasmic domain (C) were not included in the construct. The human tissue plasminogen activator (tPA) leader sequence replaced the native AMA1 signal sequence as previously described [28]. This construct was used to make recombinant E1 and E3 deleted human adenovirus serotype 5 (AdHu5) and the attenuated orthopoxvirus modified vaccinia virus Ankara (MVA) expressing AMA1. The methods used for the generation of the vectors have been previously described [32], [42]. The second generation mono-allelic AMA1 vaccines are described in Figure 4A. A final construct was designed to contain a fusion of two divergent alleles of AMA1 (3D7 and FVO) linked by a Glycine-Proline (GGGPGGG) linker. It also had the human tPA leader sequence and the TM and C terminus from the FVO strain and was human codon optimized. This construct (3483 bp in length) is described elsewhere [44] and was cloned into AdHu5 and MVA as before, as well as E1/E3 deleted chimpanzee adenovirus 63 (ChAd63, previously known as AdCh63) as described [32].

### Animals and Immunizations

Female BALB/c (H-2^d^) mice 5–6 weeks old (John Radcliffe Hospital, Oxford, UK or Harlan, UK) were used in all experiments. All procedures were performed according to the terms of the UK Animals (Scientific Procedures) Act Project License (PPL 30/2414) and Personal License (PIL 30/7792) and were approved by the University of Oxford Animal Care and Ethical Review Committee. The vaccines were diluted in endotoxin-free Dulbecco's PBS prior to immunization. Vaccines were administered intradermally (i.d.) bilaterally into the ears in a total volume of 50 µl for both recombinant adenovirus and MVA. The doses of recombinant adenovirus vaccines used were 5×10^10^ viral particles (vp), 1×10^10^ vp or 1×10^9^ vp and the dose of recombinant MVA vaccine used was always 1×10^7^ plaque forming units (pfu) [29], [44]. The interval between the prime immunization with AdHu5-AMA1 or ChAd63-AMA1 and boosting with MVA-AMA1 was 8 weeks for all the regimens.

New Zealand white rabbits were used for all rabbit experiments. The vaccines were shipped from Oxford and the immunization of rabbits and collection of sera was performed by Biogenes GmbH, Berlin, Germany (for the mono-allelic AMA1 rabbit experiment) or Agro-bio, France (for comparison of mono-allelic and bi-allelic vaccines). Rabbits were immunized i.d. or intramuscularly (i.m.) with 5×10^10^ vp of AdHu5 or ChAd63 expressing AMA1 and 5×10^8^ pfu of MVA AMA1.

### Total IgG ELISA

The antibodies induced by vaccination were measured by ELISA as previously described [28]. Briefly, recombinant PfAMA1 protein was adsorbed to 96 well Nunc-Immuno Maxisorp plates at a concentration of 2 µg/ml in PBS. 3D7 AMA1 was a kind gift from Dr Chetan Chitnis (ICGEB, New Delhi, India) and FVO AMA1 was a kind gift from Dr Mike Blackman (NIMR, London, UK). After blocking, the serum was incubated for 2 h and the bound antibodies were detected using alkaline phosphatase-conjugated goat anti-mouse (Sigma A3562) or anti-rabbit (Sigma A8025) IgG (whole molecule) diluted 1∶5000. Serum antibody endpoint titers were taken as the x-axis intercept of the dilution curve at an absorbance value 3× standard deviations greater than the OD_405_ for naive mouse serum (typical cut-off OD_405_ for positive sera = 0.15). Naïve mice sera were pooled and used as controls for all the ELISAs and they were negative against both recombinant 3D7 and FVO PfAMA1 protein. Standardized ELISAs for PfAMA1 were performed as described elsewhere [11].

### Isotype ELISA

Mouse IgG isotype ELISA assays were performed exactly as for total IgG, except antibodies were detected by incubation for 1 h at room temperature (RT) with biotinylated rat anti-mouse IgG1 or IgG2a mAbs diluted to 1 µg/ml in Phosphate Buffered Saline +0.05% Tween. Plates were washed 6× in PBS/T and then incubated for 30 min at RT with ExtrAvidin conjugated to alkaline phosphatase (Sigma) diluted 1∶5000 in PBS/T. After the incubation the plates were developed as for total IgG.

### *In vitro* Growth Inhibitory Activity (GIA) Assay

Sera from immunized animals were used to test their ability to inhibit parasite growth. The purified IgG was mixed with *P. falciparum* infected human red blood cells and the growth inhibition activity of the serum was determined by measuring parasite lactate dehydrogenase (pLDH) release after a 40–48 hour incubation period. Purified IgG was tested against 3D7, FVO, or FCR3 strains of *P. falciparum* at 5 or 10 mg/ml. The mouse and rabbit GIA assays were performed using a standardized method [10] at the GIA Reference Centre (Laboratory of Malaria and Vector Research, NIH) against both 3D7 and FVO strains of *P. falciparum* parasites. The rabbit GIA assay (Figure 3B) against the 3D7 and FCR3 strains of *P. falciparum* was undertaken at the Biomedical Primate Research Centre, The Netherlands against the 3D7 and FCR3 parasite strains following a similar protocol [21].

### Multiparameter Flow Cytometry

Cytokine secretion by peripheral blood mononuclear cells (PBMCs) and splenocytes were assayed by intracellular cytokine staining (ICS). PBMCs and Splenocytes were prepared as previously described and added to a 96-well U-bottom plate. 50 µl of Brefeldin A (GolgiPlug) at 1 µg/ml and 50 µl of peptide pools [44] diluted in complete medium at a final concentration of 1 µg/ml were added to the test wells. In the control wells GolgiPlug and medium alone were added. Cells were incubated at 37°C 5% CO_2_ for 5 h. After incubation the cells were left at 4°C overnight. The next day cells were surface stained for 30 min at 4°C with PerCP-Cy5.5-conjugated anti-mouse CD8α (clone 53-6.7) and Pacific blue (PB)-labelled anti-mouse CD4 (clone LT34) diluted in PBS with 0.1% BSA at a dilution individually assessed by titration for each antibody. Cells were then permeabilized in 100 µl 1× Cytofix/Cytoperm solution for 20 min at 4°C and then intracellularly stained for 30 min at 4°C with APC-conjugated anti-mouse IFN-γ (clone XMG1.2), FITC-conjugated anti-mouse TNFα (clone 145-2C11) and PE-conjugated anti-mouse IL-2 (clone JES6-5H4). After staining, cells were resuspended in 200 µl PBS containing 1% formalin. Stained cells were acquired using a LSRII flow cytometer (BD Biosciences) and data analyzed using FlowJo v9 (Tree Star Inc, USA), Pestle and SPICE (Mario Roederer, VRC, NIAID, NIH) software. Background responses in unstimulated cells were subtracted from the stimulated responses prior to analysis.

### Immunofluorescence Assay (IFA)

Hela cells (obtained from European Collection of Cell Cultures, UK) were grown on 22 mm coverslips in 6-well plates to about 50% confluency. 20 µl Lipofectamine 2 K and 500 µl Optimem (Invitrogen) (for each well) were mixed and incubated for 5 min at RT. 4 µg plasmid DNA of the individual constructs corresponding to the transgenic insert in recombinant adenoviruses and comprising a human CMV promoter and bovine growth hormone polyadenylation signal were gently mixed with 500 µl of Optimem (per well). The DNA-Optimem and the Lipofectamine-Optimem solutions were combined and incubated for 20 min at RT. These were then used to transfect the cells grown on the coverslips by adding 1 ml of the solution to each well. These were incubated for 3–4 h at 37°C and 5% CO_2_ in a humidified incubator and then 1 ml of Optimem was added to each well and left overnight. The plates were kept on ice and the cells were gently washed twice with ice-cold PBS. The cells were then fixed with 4% paraformaldehyde in PBS for 10 min on ice and then 20 min at RT. The cells were then washed with PBS three times over 10 min and quenched with 50 mM ammonium chloride for 5 min. After this the cells were permeabilized (if required) with 0.2% Tween-100 in PBS for 5 min and washed 3 times with PBS. If the cells were not permeabilized only the cell surface protein is accessible for immunolabelling. The cells were then blocked with 0.5% BSA in PBS for 30 min and then incubated with polyclonal mouse serum from mouse immunized with AdHu5-PfAMA1 and MVA-PfAMA1 diluted 1∶100 in PBS. After 30 min Alexa 548-conjugated anti-mouse IgG (1∶200 dilution) was added (Invitrogen, UK) and incubated for another 30 min. After the incubation the cells were washed with PBS and the cover slip was mounted using 30 µl Mowiol-DAPI (4′,6′-diamino2-phenylindole, 500 ng/ml). Slides were imaged using a Leica DMI3000B epifluorescence microscope and a Qicam (Qimaging).

The parasite immunofluorescence assay was performed according to a published protocol [66]. Cold methanol-fixed smears prepared from a synchronized culture of *P. falciparum* with fully mature schizonts were used for the assay. The slides were fixed with acetone and incubated for 30 min with a serial dilution of the post immunization serum and control serum in PBS containing 1% BSA and 0.01% sodium azide. After incubation these were washed and incubated with a 1∶80 dilution of FITC-conjugated goat anti-rabbit IgG (Dako, UK) for 30 min. The slides were immersed in a solution of 0.1% Evans blue and 0.001% DAPI and mounted on coverslips and examined microscopically as above.

### Capture ELISA

Adenovirus infectious units (i.u.) were titered by immunostaining, carried out by infection of 293 cells with serial dilutions of virus. Forty-eight hours post-infection, cells were stained with anti-hexon antibody (Cambridge Bioscience, UK) and detected with HRP-conjugated secondary antibody (Cambridge Bioscience, UK) and ImmPact DAB reagent (Vector Labs, UK). 1×10^6^ A549 cells were infected with either 1×10^7^ i.u. of AdHu5-tPA-PfAMA1 or AdHu5-N-PfAMA1. Two days later both the supernatant and the lysate were harvested separately. Briefly, rat anti-PfAMA1 monoclonal antibody 4G2 was adsorbed to 96 well Nunc-Immuno Maxisorp plates at a concentration of 100 µg/well in PBS. After blocking with 10% skimmed milk in PBS with 0.05% Tween-20 (PBS/T), the supernatant and lysate were diluted 1∶50 and 1∶500 in PBS/T and incubated for 2 h, before addition of rabbit anti-PfAMA1 polyclonal serum (diluted 1∶1000 in PBS/T) and incubation for a further 2 h. The bound antibodies were detected using alkaline phosphatase-conjugated goat anti-rabbit (Sigma A8025) IgG (whole molecule) diluted 1∶5000. The OD_405_ values were measured.

### Statistical analysis

Data were analyzed for statistical significance using GraphPad Prism v5 for windows (GraphPad Software, San Diego, CA). The normality of the data set was determined using the Kolmogorov-Smirnov one-sample test. For non-parametric data a Mann-Whitney U test was used to compare two groups and a Kruskal-Wallis test was used to compare more than two groups. Parametric data were compared either using t-test (for comparing two groups) or one-way ANOVA (for more than two groups). A post-hoc Dunnet's correction was used to compare groups to one control group, whilst a Bonferroni correction was used when all the groups were compared to each other. ELISA titers were logarithmically (log10) transformed in order to normalize the data and enable a more stringent parametric analysis. Wilcoxon signed rank test was performed to compare mean responses between paired data across two observations. Correlations were tested using Pearson rank correlation for parametric data (log-transformed data) or Spearman's rank correlation for non-parametric data. *P *≤ 0.05 was considered significant in all cases (*P≤ 0.05, ** *P *≤ 0.01 and ***P ≤ 0.001).
